# Growth-Rate Related Quantitative Trait Locus Analysis of Monokaryotic Isolates of *Grifola albicans* f. *huishuhua* (Maitake)

**DOI:** 10.3390/jof11120865

**Published:** 2025-12-05

**Authors:** Panpan Zhang, Junling Wang, Guojie Li, Shangshang Xiao, Lei Sun, Xiao Li, Jinghua Tian, Ming Li, Shoumian Li

**Affiliations:** 1 Key Laboratory of Vegetable Germplasm Innovation and Utilization of Hebei, College of Horticulture, Hebei Agricultural University, Baoding 071001, China; 2 College of Life Sciences, Hebei Agricultural University, Baoding 071001, China

**Keywords:** Maitake, single nucleotide polymorphism marker, quantitative trait locus, candidate gene

## Abstract

A genetic linkage map of *Grifola albicans* f. *huishuhua* (Maitake) is an important resource for chromosome analysis and the genetic basis of phenotypic variation determination. A total of 92 monokaryotic isolates were selected from the F1 generation of Q3-8 × Y1-18 in this study. Restriction site-associated DNA sequencing, as well as identification of single nucleotide polymorphisms (SNPs), was performed, aiming to illustrate a high-density genetic linkage map. A total of 1122 high-quality SNP markers were located on a map with a length of 1473.60 centimorgan (cM) by screening 589534 SNPs. This map covers 12 linkage groups (LGs) with an average genetic distance of 122.80 cM. Three quantitative trait loci (QTLs) related to the growth rate of *G. albicans* f. *huishuhua* strains were identified using the composite interval mapping method. These QTLs were mapped to linkage groups (LGs) as follows: LG3 (qmgv), LG4 (qmb), LG5 (qmd), LG8 (qrdm1, qrdm2), and LG10 (qmgrc1, qmgrc2, qmgrc3). The genes associated with mycelial growth rate and biomass production of these strains were identified. This information could be used for molecular marker-assisted selective breeding in *G. albicans* f. *huishuhua*.

## 1. Introduction

*Grifola albicans* f. *huishuhua* X.J. Xie et al., previously identified as *G. frondosa* in East Asia, is a well-known yet rare edible fungus with medicinal properties. This polyporoid fungus is a member of the family Meruliaceae in the order Polyporales [1,2]. It is known for its uniquely delicious flavor and is rich in a variety of bioactive substances and health benefits such as antitumor, antioxidant, hypoglycemic, and hypolipidemic properties, as well as immune-strengthening elements [3,4,5,6,7,8]. Although commercialization of this edible fungus has grown rapidly with great development potential, efforts in breeding have not kept pace with this progress.

The rapid development of molecular technology, next-generation sequencing, bioinformatics, and genomics has driven significant progress in the molecular genetic breeding of edible fungi. The edibility and health benefits of relevant varieties have been significantly improved in recent years. New varieties with efficiency improvements were bred through molecular biological technology for a number of edible fungi, such as *Agaricus bisporus* [9], *Flammulina filiformis* [10], *Lentinus edodes* [11], *Pleurotus eryngii* [12], and *Pleurotus nebrodensis* [13].

High-throughput sequencing, specifically genotyping-by-sequencing (GBS), has significantly facilitated the construction of genetic maps and the genotyping of single-nucleotide polymorphisms (SNPs) in mapping populations [14,15]. SNPs have become the preferred markers in genetic research [16,17]. GBS can simultaneously detect thousands of SNPs and corresponding genotypes with its high-throughput capability. Although the number of sequenced or re-sequenced edible fungi has increased, the GBS genotyping strategy has just been used for rapid genotyping of edible fungi in recent years [18,19]. There were no reports on the application of GBS in the genetic research of *G. albicans* f. *huishuhua*.

Quantitative Trait Loci (QTLs) refer to specific regions in the genome that control quantitative traits—traits showing continuous variation, such as growth rate, yield, and quality [20]. High-resolution genetic linkage maps are crucial for precise identification and characterization of QTLs, recombination detection, and comparative genomic analyses of candidate genes [21]. High-density genetic maps have also been demonstrated to be valuable tools in marker-assisted selection [17]. Only a few genetic maps have been constructed for widely cultivated edible fungi so far, such as *A. bisporus* [22,23], *Auricularia auricula-judae* [24], *F. filiformis* [25], *Gloeostereum incarnatum* [26], *L. edodes* [11], *P. eryngii* [27], and *Pleurotus tuoliensis* [18]. The absence of a genetic linkage map in *G. albicans* f. *huishuhua* impedes the identification of genotype-phenotype associations.

QTL mapping, which is based on a molecular genetic linkage map, can significantly enhance breeding efficiency and accuracy [28]. This approach can provide a much-needed foundation for the genetic improvement of *G. albicans* f. *huishuhua*. The primary objectives of edible fungi breeding programs include increasing the concentration of bioactive compounds and overall yield, improving quality, and enhancing disease resistance [29]. However, most of these traits are quantitatively controlled by polygenes, making conventional breeding by phenotypic selection a laborious task. Identification of QTLs for these complex traits by genetic mapping can speed up the marker-assisted selection and accelerate the breeding process [18]. The mycelial growth rate is an important factor affecting the growth cycle of *G. albicans* f. *huishuhua*. Fast-growing mycelia can colonize the medium and inhibit the growth of other microorganisms, thus improving yield and quality. Currently, there are no published reports on genes and pathways involved in the mycelial growth QTLs of this fungus.

The whole genome of *G. albicans* f. *huishuhua* was re-sequenced, and SNP markers were identified to construct a high-density genetic linkage map in this study. The mapping population consisted of monokaryotic isolate ZJ74, which represents the F1 generation from the cross. Mycelial growth measurements and genomic data were combined with QTL localization for analyzing the key growth traits. This study aims to collect regulatory information on the mycelial growth rate of *G. albicans* f. *huishuhua*, laying the foundation for molecular-assisted breeding using QTL candidate genes.

## 2. Materials and Methods

### 2.1. Fungal Strains

**Figure 1:**
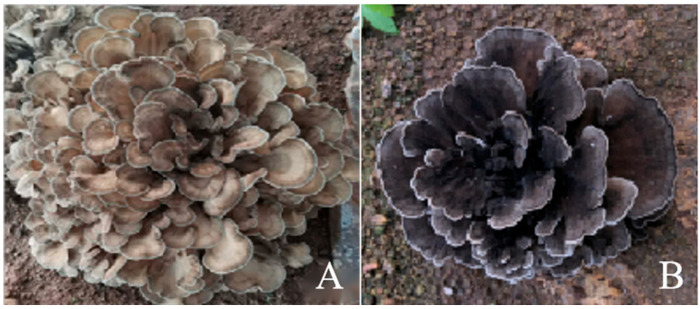
Images of parental strains. Notes: (**A**): Q3-8, (**B**): Y1-18.

Parental strains: Qianxi 3 and wild type 1; test strains: Hybrid progeny strain Q3-8 × Y1-18 (ZJ74), derived from the 8th monosporic isolate of Qianxi 3 (Q3-8) and the 18th monosporic isolate of Wild Type 1 (Y1-18). Q3-8 is a main cultivar of *G. albicans* f. *huishuhua* with the following characteristics: light color, low heat tolerance, long growth cycle, and high yield. In contrast, wild *G. albicans* f. *huishuhua* varieties are characterized by dark color, short growth cycle, and low yield (Figure 1).

After the fruiting bodies of hybrid strain ZJ74 emerged, coin-sized fruiting body pieces were suspended in a spore collector and allowed to stand for 24 h to obtain ejected spore prints. The spore suspension was diluted to 10 spores/100 μL and spread onto PDA (200 g potato infusion, 20 g glucose, 20 g agar, and 1 L distilled water) plate medium. Incubated at 25 °C for 7 days, the spores began to germinate.

### 2.2. Genome Sequencing

Monosporic strains obtained from single spore isolation of ZJ74 were used as the mapping population. Genomic DNA was extracted from strains Q3-8, Y1-18, and the monosporic strains using the CTAB method [30], and then sent to Beijing Biomarker Technologies Co., Ltd. (Beijing, China) for whole-genome resequencing.

### 2.3. SNP Marker Development

The re-sequencing results of the two parents, hybrid progeny, and test strains were analyzed by the GATK online software (https://gatk.broadinstitute.org, accessed on 1 January 2022). SNP markers were developed by comparing the second-generation resequencing data of two parent strains in this study with the *G. albicans* f. *huishuhua* whole genome (GenBank No. ASM168373v) published by Kunming University of Science and Technology. SNP genotyping was performed on the mapping population. Genotypes matching Q3-8 and Y1-18 were designated as A and B, respectively. Heterozygous genotypes were designated as “U”. Missing genotypes were labeled as “-”.

The whole genome sequence of ASM168373v1 was used as a reference, and the GATK online software calling SNPs was used to filter redundant reads using Picard based on the localization of clean reads in the reference genome (MarkDuplicates) to ensure the accuracy of the assay results. The detection of SNPs was then performed using the HaplotypeCaller (local haplotype assembly) algorithm of GATK, where each sample was first generated individually as a gVCF and then as a population joint-genotype. Final filtering was performed, and the final set of variant loci was obtained.

The variation results were strictly filtered to ensure the reliability of the variation results. The main filtering parameters were as follows: (1) SNPs within 5 bp of the INDEL and those within 10 bp of the adjacent INDEL were filtered out based on the subroutine vcfutils.pl (varFilter-w5-W10) in BCFtools, (2) ClusterSize 2 cluster WindowSize 5, (3) QUAL < 30, (4) QD < 2.0, (5) MQ < 40, (6) FS > 60.0, (7) Other variant filtering parameters were processed with the default values specified by GATK.

To ensure the quality of genetic mapping, polymorphic SNP markers were filtered according to the following rules: (1) filtering parents for sequencing depth below 15 X, (2) completeness filtering, (3) biased segregation marker filtering.

### 2.4. Construction of Genetic Linkage Map

A genetic linkage map was constructed with MSTmap [31] software (http://www.cs.ucr.edu/~yonghui/mstmap.html, accessed on 2 September 2025) with the following parameters: a cut-off *p*-value of 0.001, a missing threshold of 0.9, and the Kosambi function.

### 2.5. Determination of Mycelial Growth and Biomass

All mononuclear isolates in the targeted population were activated on PDA solid medium, and the mycelial growth rate was measured. A 5 mm mycelial plug was taken from the activated culture using a puncher and inoculated onto PDA medium. Incubated in a 25 °C incubator, timing started when the mycelial plug germinated. A line was drawn 4 days later, denoted as the “initial line”; after continued incubation for another 6 days, a second line was drawn, denoted as the “final line”. The distance between the two lines was then measured. Four measurements were taken per Petri dish, with five biological replicates set for each strain. Finally, the daily mycelial growth rate was calculated. When measuring the fungal growth rate in chestnut sawdust culture medium (CW), activated mycelial blocks with a diameter of 8 mm should be punched out using a puncher and transferred into test tubes containing CW. When the mycelium grows to the 17th day, it is marked as the starting line of mycelial growth, and after 15 days, it is marked as the endpoint. The distance between the two lines was measured, and four readings were taken for each test tube to calculate the mycelial growth rate. When measuring fungal biomass, potato glucose liquid medium (PDL) is used. Place the activated bacterial blocks into a 150 mL triangular flask containing 30 mL PDL and culture for 15 days (25 °C, dark conditions). Measurement of Mycelial Biomass: The mycelia, from which the medium has been filtered out, are dried and weighed.

Mycelial density (MD), regularity degree of mycelia (RDM), and mycelial growth vigor (MGV) were evaluated by visual observation. These were carried out in 9 cm flat dishes containing PDA medium. MD was rated as sparse (0), dense (1), denser (2), and extremely dense (3). Mycelial edge neatness was expressed as irregular (0), relatively neat (1), neat (2), and extremely neat (3). The MGV was expressed as weak (0), stronger (1), strong (2), and extremely strong (3).

All the above measurements were repeated five times, and the results were analyzed with IBM SPSS (2024) for mean, standard deviation, coefficient of variation, and correlation analyses (Shapiro–Wilk test and Kolmogorov–Smirnov test), significance analyses, etc. Diagrams were plotted with OriginPro 2024 SR1.

### 2.6. QTL Localization and Gene Annotation

Conduct QTL mapping analysis using R/qtl, and map traits through the composite interval mapping method. Set the threshold by performing 1000 permutation tests (PT tests). First, consider the LOD threshold corresponding to a 0.99 confidence level; if no mapping interval is found, consider the LOD threshold corresponding to a 0.95 confidence level. If there is still no mapping interval, consider the threshold of a 0.90 confidence level. If no results are obtained yet, disregard the results of the PT test and manually lower the threshold to 3.0. If there is no interval at 3.0, lower it to 2.5; if there is still no interval at 2.5, lower it to 2.0. Analyze the statistically summarized data of each trait using MapQTL 6.0 software.

Compare the mapped regions associated with mycelial growth rate and biomass with the reference genome to obtain the gene information contained in the sequences of the mapped regions. Then, gain insights into their functions through GO functional annotation, KEGG enrichment analysis, and Swiss-prot annotation, and identify the main physiological and biochemical metabolic pathways as well as signal transduction pathways that these genes are involved in.

## 3. Results

### 3.1. SNP Marker Screening

The 92 monokaryotic isolates of *G. albicans* f. *huishuhua* were re-sequenced using the second-generation sequencing method, obtaining 106.54 Gb of clean data and 92.52% Q30. The average sample-to-reference genome alignment rate was 90.07%, the average coverage depth was 20 X, and the genome coverage was 86.44%.

A total of 589,534 SNPs were obtained after detection and filtering. Of the 364,141 SNPs obtained that could be successfully typed, 223,079 SNP markers matched the aa × bb genotype. To ensure the quality of genetic mapping, a set of 1122 high-quality SNP markers was finally obtained after filtering polymorphic SNPs and can be used for mapping.

### 3.2. Genetic Linkage Map

**Figure 2:**
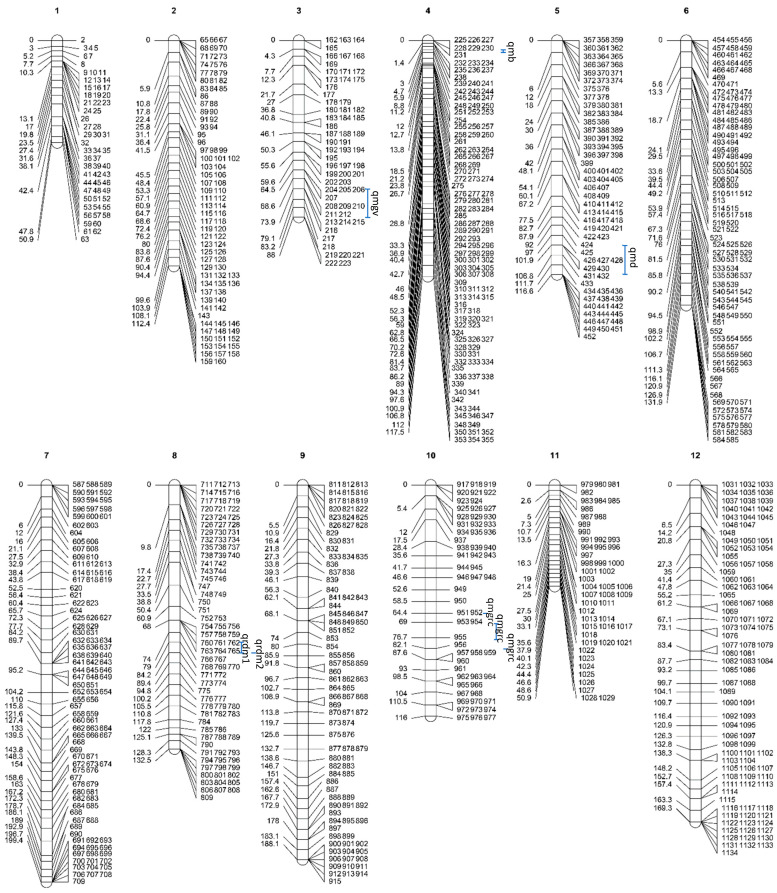
High-density genetic linkage map of *G. frondosa*.

The MSTmap software (https://mstmap.org, accessed on 1 February 2022) was applied to construct 12 chain clusters of 1122 high-quality SNP markers. The high-density genetic linkage map of *G. albicans* f. *huishuhua* was obtained by analyzing the linear arrangement of markers within linkage clusters using HighMap software v2.0 and estimating the genetic distance between adjacent markers, as shown in Figure 2.

The total map distance (total length of the map) for the 12 genetic linkage groups (LGs) was 1473.60 cM (Table 1), with an average length of 122.8 cM. The number of markers in the 12 linkage groups ranged from 51 to 132, with an average of 93.5 and an average genetic distance between markers of 1.31 cM. The longest linkage group was LG7, with a length of 199.42 cM, consisting of 123 markers. The maximum gap spacing ranged from 3.56 cM to 11.62 cM, with an average spacing of 8.99 cM, of which LG11 had a gap map spacing of less than 5 cM. Using a χ^2^ test (*p* < 0.05) on 1122 markers, 1093 markers met the 1:1 condition, and 29 did not. The latter accounted for 2.58% of the total number of markers; LG9 and LG12 had segregation aberrations.

**Figure 3:**
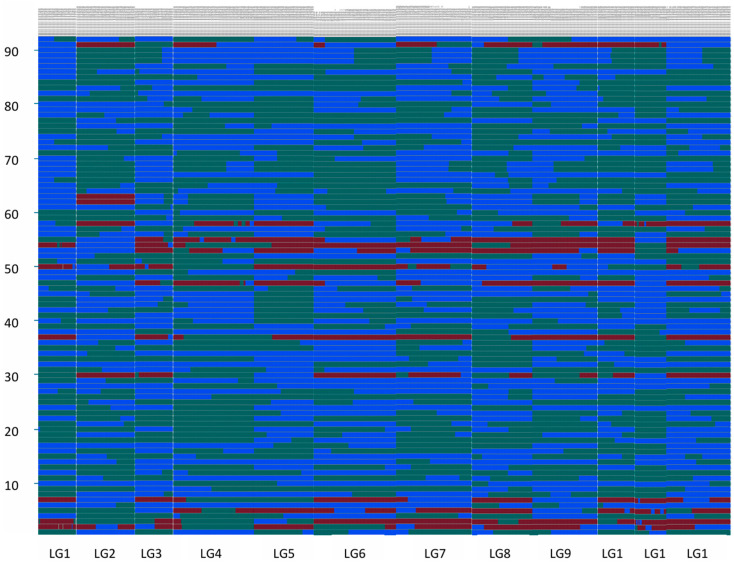
Visualization of individual genotypes. Note: the abscissa corresponds gene marker location of each linkage group, and the ordinate is the mapping individual. The green fragment originated from parent Q3-8. The blue fragment originated from parent Y1-18. The red fragment was derived from hybrid progeny.

SNP markers were unevenly distributed in the linkage population (Figure 2), and for each LG, many individuals inherited the full biparental type, as shown in Figure 3. No more than two crossovers occurred in LG1 for the remaining 90 individuals. Exceptions are 1-3/1-54 with three/five crossovers occurring correspondingly. Of these 32 individuals in LG1, including 1-1, 1-5, had entire gene segments inherited from Q3-8. No crossovers were observed. A total of 44 individuals in LG1, including 1-4, 1-6, had entire gene segments inherited from Y1-18. No crossovers were observed. Similar patterns of inheritance were observed in other individuals. However, none is inherited from a single parental line for all linkage groups. Among all individuals, the minimum proportion of parental genotypes for Q3-8 was 11.32%, the minimum proportion of parental genotypes for Y1-18 was 23.08%, the maximum number of crosses in individuals was 25, the minimum number of crosses in individuals was 5, and the average number was 11.

### 3.3. Mycelial Growth Rate and Biomass

**Figure 4:**
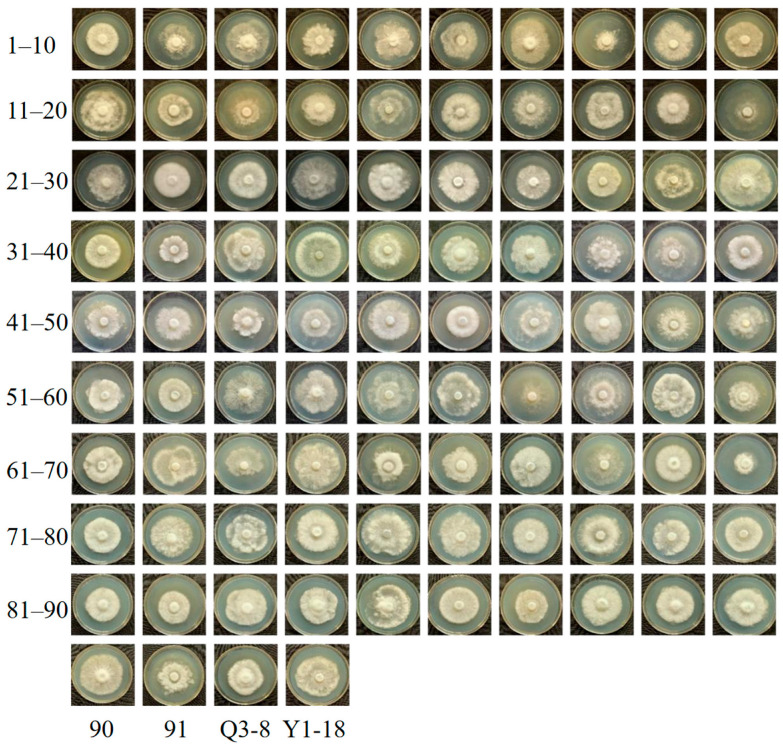
Two parents and the mycelial morphology of the 94 monokaryotic isolates of *G. albicans* f. *huishuhua.* Note: The first row of pictures from left to right are 1, 2, 3 … 10, and the second row of pictures from left to right are 11, 12, 13 … 20, and so on, with the parent strains being Q3-8, Y1-18.

Phenotypic traits such as mycelial growth rate (MGR), mycelial biomass (MB), mycelial density (MD), regularity degree of mycelia (RDM), and mycelium growth vigor (MGV) of the mapping populations varied significantly (Table 2). Colony characteristics also varied greatly (Figure 4). The MGR in the wood chip medium of Q3-8 was 3.860 mm/d and 4.056 mm/d for Y1-18. The MGR of the 92 monokaryotic F1 isolates was 2.768–4.953 mm/d, with an average of 3.779 mm/d and a coefficient of variation of 0.115 (Table 2, Supplementary Table S1). The mycelial biomass of Q3-8 was 0.032 g/d, and Y1-18 was 0.025 g/d. Among the 92 monosporous F1 strains, the mycelial biomass growth rate ranged from 0.017 to 0.069 g/d, with an average of 0.039 g/d. The average rate of mycelial linear extension was 3.779 mm/d (Table 2, Supplementary Table S2). The coefficients of variation for MB, RDM, and MGV were 0.502, 1.061, and 0.584, respectively. The larger coefficients of variation indicated that the trait was separate and discrete; the relationship between the markers and the trait could be better estimated, which was conducive to QTL localization.

**Figure 5:**
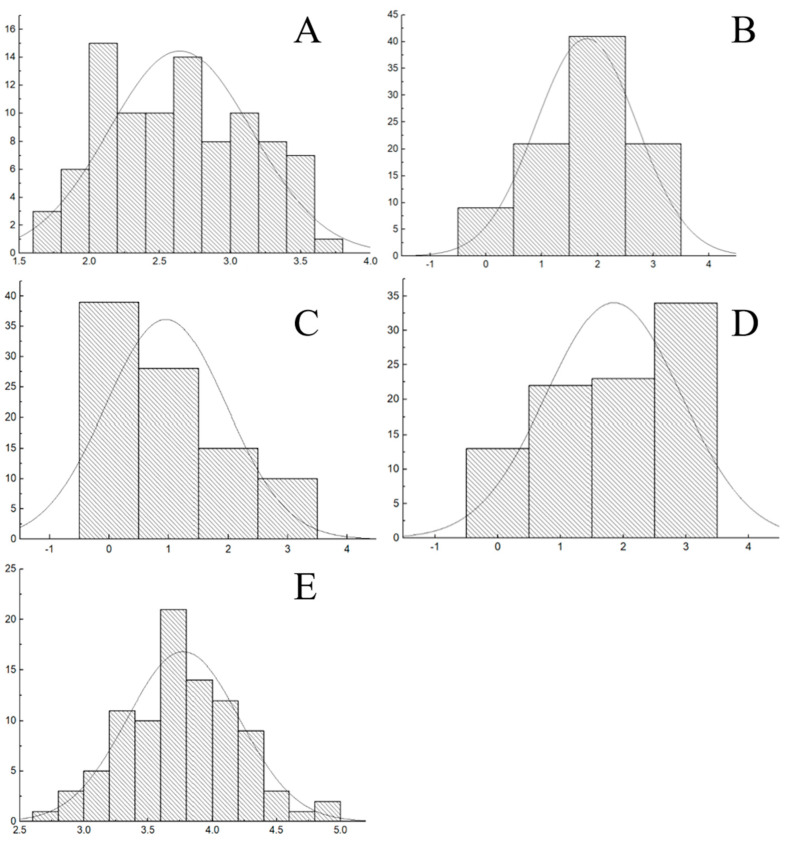
Distribution of agronomic characters in mononuclear mycelia. Notes: (**A**) growth rate distribution of monokaryon mycelium on PDA medium; (**B**) density distribution of monokaryon mycelium; (**C**) regularity degree distribution of monokaryon mycelium; (**D**) growth vigor distribution of monokaryon mycelium; (**E**) growth rate distribution of monokaryon mycelium on CW medium.

The Kolmogorov–Smirnov and Shapiro–Wilk tests for the mycelial growth rate, biomass, mycelial density, mycelial neatness, and mycelium growth vigor of the mapping population did not conform to a normal distribution (*p* < 0.05). These two tests for mycelial growth rate and biomass were positively skewed with a skewness of 0.088 and 0.494 and a kurtosis of 0.148 and −0.119, respectively (Figure 5). However, these two tests were negatively skewed with a skewness of −0.419, −0.723, and −0.388 and a kurtosis of −0.523, −0.619, and −1.619.

### 3.4. QTL Location of Mycelial Growth Indicators

QTLs of five agronomic traits in *G. albicans* f. *huishuhua* were mapped with composite interval mapping and were localized to linkage groups LG3, LG4, LG5, LG8, and LG10. The integration of contrasting genomes and genetic maps enabled us to screen putative candidate genes at QTL. By comparing the eight QTLs with genomic data of this species published on GenBank, a total of 280 candidate genes were obtained, of which 192 of these genes were annotated to at least one of the databases (COG, GO, KEGG, Swissprot, and NR), as shown in Table 3. A total of 192 genes were compared with the NR database, and 46 species related to *G. albicans* f. *huishuhua* were obtained (Table 4), which was similar to the transcriptome sequencing results of Nie et al. [29].

The three related motifs (qmgrc1, qmgrc2 and qmgrc3) associated with MGRC were detected on LG10, which were tightly linked to SNP952, SNP953-955 and SNP956, respectively. The mapping distances were 98.507 cM, the LOD threshold was 2.500, and the maximum LOD value was 2.695, which reached a highly significant level. The contribution to phenotypic variation was 11.80% with an additive effect value of −0.157. The QTL affecting MB was located on LG4, which was closely associated with SNP244-246. Corresponding data values are a map distance of 8.840 cM, a LOD threshold of 2.987, and a maximum LOD value of 3.023, reaching an extremely significant level, and contributing to the phenotypic variance of 7.778%, with an additive effect value of 0.004. The locus qmd distribution of loci related to MD in the mapping population is on LG5. It was closely linked to SNP429-447, with a mapping distance of 116.624 cM, an LOD threshold of 3.00, and a maximum LOD value of 3.006. The highly significant level contributed to the phenotypic variance of 6.275% and had an additive effect value of 0.237. Two related loci (qrdm1 and qrdm2) associated with RDM were detected on LG8, and were closely linked to SNP770-771 and SNP772, respectively. The mapping distances were 105.525 cM, the LOD threshold was 2.00, and the maximum LOD value was 2.113. This reached an extremely significant level, with a contribution to phenotypic variation of 6.234%, and the additive effect value was −0.266. The locus qmgv associated with MGV in the mapping population was located in LG3, which was closely linked to SNPs 216 to 220. The map spacing was 88.016 cM, with an LOD threshold of 3.375, and a maximum LOD value of 3.566 at a highly significant level. This contributes 7.552% to the phenotypic variance and to an additive effect value of 0.311.

### 3.5. Locus Analysis

The physical location of the QMGV locus was 2.1, starting position was 3,011,430, stop position was 3,070,889, and gene size was 0.06 M. Twenty-four candidate genes were found, among which 11 were annotated. The physical location of the QMB locus was 27.1, the starting position was 68,279, the ending position was 193,511, and the size of the gene was 0.13 M. Fifty-three candidate genes were found, among which 38 were annotated. The physical location of the QMD locus was 15.1, the starting position was 902,526, the ending position was 1,283,453, and the size of the gene was 0.38 M. One hundred and thirty-six candidate genes were found, of which 93 were annotated. The rdm1.1 and q38.9 were the starting and ending positions of q328 and Q38, respectively. No candidate gene was found in qrdm2, while a candidate gene was found in qrdm1 and annotated into the NR database. The physical location of the qmgrc2 locus was 43.1, the starting position was 34,078, the ending position was 196,166, and the gene size was 0.16 M. A total of 65 candidate genes were found, of which 48 were annotated. The physical positions of qmgrc1 and qmgrc3 were 23.1, and the starting and ending positions of qmgrc1 were 48,150. The starting and ending positions of qmgrc3 were 45,701. No candidate gene was found in qmgrc1, and one candidate gene was found in qmgrc3 and annotated (Table 5).

## 4. Discussion

The construction of genetic linkage maps provides a basis for quantitative trait locus (QTL) mapping and breeding through marker-assisted selection (MAS), which is important for genetic research [32]. QTL mapping is helpful for genetic improvement of quantitative traits. Some edible fungi, such as *A*. *bisporus* [33,34,35] or *F. velutipes* [36], have completed the construction and mapping of genetic linkage maps for important agronomic quantitative traits. At present, genetic linkage map construction through resequencing has become a more efficient and cost-effective method than traditional methods [37]. The resequencing population of edible fungi is usually carried out based on single-spore isolates of hybrid offspring. Only one allele of each gene is expressed in a dominant manner, thus creating a better correspondence between phenotype and genotype [38]. These methods are particularly suitable for studying QTLs related to hyphal growth rate.

In this study, we successfully obtained a total of 92 monokaryotic isolates from *G. albicans* f. *huishuhua*. A genetic linkage map of *G. frondosa* that included 12 linkage groups was constructed, with the longest group not exceeding 200 cM and the shortest not less than 50 cM. These 12 linkage groups probably correspond to the 12 chromosomes obtained by our group previously [39], which is similar to the results in *Hericium erinaceus* [19]. The occurrence of marker segregation distortion is common in the construction of genetic linkage maps for plants and fungi. The distorted segregation regions in LG9 and LG12 may be caused by genetic factors related to spore germination and growth. The results showed that the frequency of offspring exchange ranged from 2 to 25 times. The individuals were more influenced by both wild parents than the main cultivar, which was more than twice that of the cultivar. The reason may be that wild varieties have stronger genetic capability. Some unique traits were found in individuals with the absence of both parents. A number of strains showed heterozygosity in the QTL mapping. It is due to impurities in monokaryotic isolates.

There are three QTLs in the genetic map of gray tree flowers that affect the growth rate of single-strain hyphae, namely qmgrc1, qmgrc2, and qmgrc. These QTLs contain 66 genes, of which six genes are enriched in pathways related to nutrient metabolism. A total of 17 enzymes related to fungal stress resistance were identified in gene prediction. This is similar to the research results of *F*. *velutipes*, where the QTL (qMGRP1-LG7) affecting the growth of single-strain hyphae covers a genome sequence of 398 kilobase pairs (kb) and contains 134 genes. According to the Kyoto Encyclopedia of Genes and Genomes (KEGG) analysis, six genes related to metabolism were enriched [23]. Three QTLs controlling hyphal growth rate were identified in *L*. *edodes*. Simultaneously identifying three candidate genes regulating hyphal growth rate (Le TDC, Le CH, and Le CP) [40]. Therefore, in *G. albicans* f. *huishuhua*, candidate genes can be predicted, fine-scale gene mapping can be carried out, and gene function research can be conducted.

The eight QTLs related to hyphal growth detected from G are closely related to the markers on the linkage map. Three of them are related to hyphal growth rate, two are related to hyphal density, one is related to hyphal biomass, one is related to both hyphal density and hyphal growth, and one is related to hyphal elongation. Similarly to research on *L. edodes* [41] and *F. velutipes* [32]. QTL analysis of *F*. *velutipes* revealed a gene locus, qMGRP1-LG7, that controls hyphal growth rate. This locus contains 134 genes and is enriched with pathways and genes related to substance metabolism. Two QTLs controlling the growth rate of mycelium in *Auricularia polytricha* on sawdust medium are located on LG10 and LG12 [33]. A QTL was detected to control the hyphal growth rate of a single strain of *F*. *filiformes* on the cultivation substrate [32]. The study on *Auricularia heimuer* also proved that using mycelial growth rate as a selection feature to cultivate varieties with different growth cycles is unreliable [42]. But the yield is closely related to the growth cycle, and these two traits are negatively correlated. Similar results were observed in *A. bisporus* [34], *Pleurotus ostreatus* [43], and *P. eryngii* [28]. This provides a scientific basis for cultivating high-yield varieties with different growth cycles in *A*. *bisporus* [44]. These closely linked markers can be considered for molecular marker-assisted selection of excellent strains with hyphal stage-specific traits. This also proves that using mycelial growth rate as a selection feature to cultivate varieties with different growth cycles is unreliable. However, there is no correlation between these two traits, resolving the controversy in genetic linkage analysis of these traits. Search for two to four wild varieties of *G*. *frondosa* and cross them with the mycelia of 92 single strains. This is for QTL mapping, aiming to identify genes related to mycelial growth and agronomic traits in the mycelia of dual-strain hybrids and to reconstruct the genetic system of *G. albicans* f. *huishuhua*.

## Figures and Tables

**Table 1:** Characteristic index of *G. albicans* f. *huishuhua*. genetic linkage group.

Linkage Group	Number of Markers	Map Distance (cM)	Average Distance (cM)	Distance Max Gap (cM)	Gap <5 cM	Number of Distorted Markers	Partial Isolation Tag %
LG1	62	50.90	0.82	6.50	96.72%	1	1.61%
LG2	96	112.41	1.17	7.01	93.68%	0	0.00%
LG3	62	88.02	1.42	9.74	88.52%	2	3.23%
LG4	131	117.48	0.90	8.78	96.15%	3	2.29%
LG5	96	116.62	1.21	10.30	84.21%	0	0.00%
LG6	132	131.91	1.00	9.91	92.37%	0	0.00%
LG7	123	199.42	1.62	8.99	79.51%	0	0.00%
LG8	99	132.53	1.34	11.62	81.63%	0	0.00%
LG9	105	188.09	1.79	10.19	72.12%	13	12.38%
LG10	61	115.98	1.90	10.94	71.67%	0	0.00%
LG11	51	50.93	1.00	3.56	100.0%	0	0.00%
LG12	104	169.31	1.63	10.30	78.64%	10	9.62%
Totals	1122	1473.60	-	-	-	29	2.58%

**Table 2:** Statistical analysis of the agronomic traits of mononuclear mycelia.

Phenotype	Q3-8	Y1-18	Mean	MSD	RMSD	Min	Max	Coefficient of Variation
MGRC (mm/d)	3.860	4.056	3.779	0.188	0.433	2.768	4.953	0.115
MB (g/d)	0.032	0.025	0.039	0.000	0.013	0.017	0.069	0.333
MD	3	2	1.804	0.818	0.905	0	3	0.502
RDM	2	1	0.957	1.031	1.015	0	3	1.061
MGV	2	2	1.848	1.163	1.079	0	3	0.584

**Table 3:** Statistical analysis of the locus annotation of each trait.

Trait	Locus	Physical Location	Gene Size (M)	Gene Number	Annotation					All Anno
					COG	GO	KEGG	Swissprot	NR	
MGV	qmgv	2.1	0.06	24	1	3	2	7	11	11
MB	qmb	27.1	0.13	53	11	20	14	24	38	38
MD	qmd	15.1	0.38	136	16	20	15	29	93	93
RDM	qrdm1	127.1	0	1	0	0	0	0	1	1
	qrdm2	9.1	0	0	0	0	0	0	0	0
MGRC	qmgrc1	23.1	0	0	0	0	0	0	0	0
	qmgrc2	43.1	0.16	65	10	15	6	17	48	48
	qmgrc3	23.1	0	1	0	1	0	1	1	1
Totals	-	-	-	280	38	59	37	78	192	192

**Table 4:** Relevant species and numbers of genes obtained from the NR database.

Species	Numbers of Genes
*Trametes cinnabarina*	29
*Trametes versicolor* FP-101664 SS1	28
*Ceriporiopsis subvermispora* B	22
*Fibroporia radiculosa*	19
*Dichomitus squalens* LYAD-421 SS1	11
*Piloderma croceum* F 1598	8
*Galerina marginata* CBS 339.88	7
*Fusarium fujikuroi* IMI 58289	4
*Sphaerobolus stellatus* SS14	3
*Jaapia argillacea* MUCL 33604	3
*Coprinopsis cinerea okayama* 7#130	2
*Hydnomerulius pinastri* MD-312	2
*Postia placenta* Mad-698-R	2
*Fomitopsis pinicola* fp-58527 SS1	2
*Plicaturopsis crispa* fd-325 SS-3	2
Other	31

**Table 5:** Relevant species and numbers of genes obtained from the NR database.

Trait	Loci	Linkage Group	Gene Function
MGV	*qmgv*	LG3	COG: Energy Production and Conversion (Category C), Signal Transduction (Catgory T); GO: Oxidoreductase activity, Stress response; KEGG: Glycolysis/Gluconeogenesis, Fatty acid degradation
MB	*qmb*	LG4	COG: Chromatin Structure (Category B), Cell Wall Biosynthesis (Category M); GO: Cell component organization, Catalytic activity; KEGG: N-Glycan Biosynthesis, Amino acid metabolism
MD	*qmd*	LG5	COG: Carbohydrate Transport (Category G), Transporter Proteins (Category P); GO: Transporter activity, Carbohydrate metabolism; KEGG: Sugar Transport Pathway, Steroid biosynthesis
RDM	*qrdm1*	LG8	COG: Secondary Metabolism (Category Q), Energy Production (Category C); GO: Hydrolase activity, Metabolic process; KEGG: Terpenoid Backbone Biosynthesis, Cellulose degradation pathway
	*qrdm2*		
MGRC	*qmgrc1*	LG10	COG: Signal Transduction (Category T), General Function Prediction (Category R); GO: Signal transduction, Growth regulation; KEGG: MAPK signaling pathway, Cell cycle regulation
	*qmgrc2*		
	*qmgrc3*		

## Data Availability

The original data presented in the study are openly available in NCBI at https://ftp.ncbi.nlm.nih.gov/genomes/genbank/fungi/Grifola_frondosa/latest_assembly_versions/GCA_001683735.1_ASM168373v1/ (accessed on 25 November 2025).

## Supplementary Materials

The following supporting information can be downloaded at https://www.mdpi.com/article/10.3390/jof11120865/s1, Table S1: MGRC statistical analysis of monokaryon mycelium on sawdust medium, Table S2: Growing rate statistical analysis of MGRP of monokaryon mycelium on PDA medium.

## Abbreviations

The following abbreviations are used in this manuscript:

MB	Mycelial biomass
MD	Mycelial density
MGRC	Mycelial growth rate (CW)
MGV	Mycelial growth vigor
RDM	Regularity degree of mycelia

## References

[1] Chen M.M. (2002). Forest Fungi Phytogeography: Forest Fungi Phytogeography of China, North America, and Siberia and International Quarantine of Tree Pathogens.

[2] Xie X.J., Wu F., Li S.M., Vlask J., Zhang X., Tian J.H., Li M., Li G.J. (2024). Revisin of the scientific name for edible and medicinal fungus huishuhua (*Maitake*) in China. Mycosystema.

[3] Wang Y., Shen X., Liao W., Fang J., Chen X., Dong Q., Ding K. (2014). A heteropolysaccharide, L-fuco-D-manno-1,6-α-D-galactan extracted from *Grifola frondosa* and antiangiogenic activity of its sulfated derivative. Carbohydr. Polym..

[4] Li Q., Zhang F., Chen G., Chen Y., Zhang W., Mao G., Zhao T., Zhang M., Yang L., Wu X. (2018). Purification, characterization and immunomodulatory activity of a novel polysaccharide from *Grifola frondosa*. Int. J. Biol. Macromol..

[5] He X., Wang X., Fang J., Chang Y., Ning N., Guo H., Huang L., Huang X., Zhao Z. (2017). Polysaccharides in *Grifola frondosa* mushroom and their health promoting properties: A review. Int. J. Biol. Macromol..

[6] Chen X., Ji H., Xu X., Liu A. (2019). Optimization of polysaccharide extraction process from *Grifola frondosa* and its antioxidant and anti-tumor research. J. Food Meas. Charact..

[7] Kou L., Du M., Liu P., Zhang B., Zhang Y., Yang P., Shang M., Wang X. (2019). Anti-diabetic and anti-nephritic activities of *Grifola frondosa* mycelium polysaccharides in diet-streptozotocin-induced diabetic rats via modulation on oxidative stress. Appl. Biochem. Biotechnol..

[8] Zhang S.S., Li X., Li G.J., Huang Q., Tian J.H., Wang J.L., Li M., Li S.M. (2023). Genetic and molecular evidence of a tetrapolar mating system in the edible mushroom *Grifola frondosa*. Acta Edulis Fungi.

[9] Chauhan S., Kapoor S., Thakur S. (2012). RAPD marker assisted development of improved strains of *Agaricus bisporus* (Lange) Sing. J. Pure Appl. Microbiol..

[10] Wu T., Ye Z., Guo L., Yang X., Lin J. (2018). De novo transcriptome sequencing of *Flammulina velutipes* uncover candidate genes associated with cold-induced fruiting. J. Basic Microb..

[11] Gong W., Li L., Zhou Y., Bian Y., Kwan H., Cheung M., Xiao Y. (2018). Detection of quantitative trait loci underlying yield-related traits in shiitake culinary-medicinal mushroom, *Lentinus edodes* (Agaricomycetes). Int. J. Med. Mushrooms.

[12] Liu W.X., Wang T.T., Tang Y.Q., Liang Y.J. (2025). Evaluation of *Pleurotus eryngii* germplasm resources and breeding of excellent hybrid strains. China Cucurbits Veg..

[13] Liu S., Wu X., Liu X., Ke B. (2016). Correlation between mating compatibility and the phylogenetic relationship of a rare edible mushroom, *Pleurotus nebrodensis*, with different Pleurotus species. Int. J. Agric. Biol..

[14] Galpaz N., Gonda I., Shem-Tov D., Barad O., Tzuri G., Lev S., Fei Z., Xu Y., Mao L., Jiao C. (2018). Deciphering genetic factors that determine melon fruit-quality traits using RNA-Seq-based high-resolution QTL and eQTL mapping. Plant J..

[15] Paudel D., Kannan B., Yang X., Harris-Shultz K., Thudi M., Varshney R., Altpeter F., Wang J. (2018). Surveying the genome and constructing a high-density genetic map of napiergrass (*Cenchrus purpureus* Schumach). Sci. Rep..

[16] Lu M., Li J., Sun X., Zhao D.-Q., Zong H., Tang C., Li K., Zhou Y., Xiao J. (2024). Genotyping single nucleotide polymorphisms in homologous regions using multiplex kb level amplicon capture sequencing. Mol. Genet. Genom..

[17] Jafer M., Rajat A., Ramesh B., Siva K. (2012). SNP markers and their impact on plant breeding. Int. J. Plant Genom..

[18] Gao W., Qu J., Zhang J., Sonnenberg A., Chen Q., Zhang Y., Huang C. (2018). A genetic linkage map of *Pleurotus tuoliensis* integrated with physical mapping of the de novo sequenced genome and the mating type loci. BMC Genom..

[19] Gong W., Xie C., Zhou Y., Zhu Z., Wang Y., Peng Y. (2020). A Resequencing-based ultradense genetic map of *Hericium erinaceus* for anchoring genome sequences and identifying genetic loci associated with monokaryon growth. Front. Microbiol..

[20] Gong W.B., Xiao Y., Zhou Y., Bian Y.B. (2011). Research progress on QTL mapping in edible fungi. Acta Hortic. Sin..

[21] Han K., Jeong H., Yang H., Kang S., Kwon J., Kim S., Doil C., Kang B. (2016). An ultra-high-density bin map facilitates high-throughput QTL mapping of horticultural traits in pepper (*Capsicum annuum*). DNA Res..

[22] Foulongne-Oriol M., Spataro C., Cathalot V., Monllor S., Savoie J.-M. (2010). An expanded genetic linkage map of an intervarietal *Agaricus bisporusvar*. bisporus×A. bisporus var. burnettii hybrid based on AFLP, SSR and CAPSmarkers sheds light on the recombination behaviour of the species. Fungal Genet. Biol..

[23] Foulongne-Oriol M., Rodier A., Rousseau T., Savoie J. (2012). Quantitative trait locus mapping of yield-related components and oligogenic control of the cap color of the button mushroom, *Agaricus bisporus*. Appl. Environ. Microbiol..

[24] Lu L., Yao F., Wang P., Fang M., Zhang Y., Zhang W., Kong X., Lu J. (2017). Construction of a genetic linkage map and QTL mapping of agronomic traits in *Auricularia auricula-judae*. J. Microbiol..

[25] Han X., Li Y.N., Zhao C., Liu Y., Du M., Huang Q., Xu W., Xie B.G. (2019). Growth-rate related QTL analysis of *Flammulina filiformis* monokaryotic isolates. Mycosystema.

[26] Jiang W.Z., Yao F.J., Lu L.X., Fang M., Wang P., Zhang Y.M., Meng J.J., Lu J., Ma X.X., He Q. (2021). Genetic linkage map construction and quantitative trait loci mapping of agronomic traits in *Gloeostereum incarnatum*. J. Microbiol..

[27] Im C.H., Park Y.H., Hammel K.E., Park B., Kwon S.W., Ryu H., Ryu J.S. (2016). Construction of a genetic linkage map and analysis of quantitative trait loci associated with the agronomically important traits of *Pleurotus eryngii*. Fungal Genet. Biol..

[28] Gong W.B., Liu W., Lu Y.Y., Bian Y.B., Zhou Y., Kwan H.S., Cheung M.K., Xiao Y. (2014). Constructing a new integrated genetic linkage map and mapping quantitative trait loci for vegetative mycelium growth rate in *Lentinula edodes*. Fungal Biol..

[29] Nie W.Q., Wu T.X., Zhong M., Lu H.-Y. (2017). Transcriptome sequencing and analysis of *Grifola frondosa* mycelia. Food Sci..

[30] Du X.H., Zhao Q., Xia E.H., Gao L.Z., Richard F., Yang Z.L. (2017). Mixed-reproductive strategies, competitive mating-type distribution and life cycle of fourteen black morel species. Sci. Rep..

[31] Voorrips R.E. (2002). MapChart: Software for the graphical presentation of linkage maps and QTLs. J. Hered..

[32] Li B. (2009). Genetic Linkage Map and Quantitative Trait Loci Controlling Vegetative Growth Rate in the Edible Basidiomycete *Flammulina velutipes*. Master’s Thesis.

[33] Lu X.C. (2015). Construction of a Genetic Linkage Map and QTL Mapping for Mycelial Growth Rate of Monokaryon in *Auricularia polytricha*. Master’s Thesis.

[34] Foulongne-Oriol M. (2012). Genetic linkage mapping in fungi: Current state, applications, and future trends. Appl. Microbiol. Biotechnol..

[35] Kerrigan R.W., Royer J.C., Baller L.M., Kohli Y., Horgen P.A., Anderson J.-B. (1993). Meiotic behavior and linkage relationships in the secondarily hompthallic fungus *Agaricus bisporus*. Genetics.

[36] Zhang G.Z. (2011). The construction and application of genetic linkage map based on the genome sequencing of *Flammulina velutipes*. Master’s Thesis.

[37] Foulongne-Oriol M., Rocha-de Brito M., Cabannes D., Clément A., Spataro C., Moinard M., Souza-Dias E., Callac P., Savoie J. (2016). The genetic linkage map of the medicinal mushroom *Agaricus subrufescens* reveals highly conserved macrosynteny with the congeneric species *Agaricus bisporus*. 3G-Genes Genom. Genet..

[38] Au C., Cheung M., Wong M., Chu A., Law P., Kwan H. (2013). Rapid genotyping by low-coverage resequencing to construct genetic linkage maps of fungi: A case study in *Lentinula edodes*. BMC Res. Notes.

[39] Terashima K., Matsumoto T., Hayashi E., Fukumasa-Nakai Y. (2002). A genetic linkage map of *Lentinula edodes* (shiitake) based on AFLP markers. Mycol. Res..

[40] Lin F.X., Li H.S., Huang J., Tan Q., Bao D.P. Construction of Genetic Linkage Map and Analysis of QTL for *Lentinula edodes* Using SNP Molecular Markers. Proceedings of the Abstracts of the 2015 Academic Annual Conference of the Chinese Mycological Society.

[41] Gong W.B., Liu K.F., Li X.R., Zhang L., Shen N., Bian Y.B., Xiao Y. (2022). QTL mapping reveals mating type gene LeHD1 regulating mycelial growth in shiitake mushroom, *Lentinula edodes*. Sci. Hortic..

[42] Lu L.X., Yao F.J. (2018). Construction of Genetic Linkage Map and QTL Mapping of *Auricularia polytricha*. Proceedings of the 2018 Annual Academic Conference of Mycological Society of China.

[43] Larraya L.M., Alfonso M., Pisabarro A.G., Ramírez L. (2003). MappDing of genomic regions (quantitative trait loci) controlling production and quality in industrial cultures of the edible basidiomycete *Pleurotus ostreatus*. Appl. Environ. Microbiol..

[44] Gao W., Baars J.J.P., Maliepaard C., Richard G.F., Visser R.G.F., Zhang J.X., Sonnenberg A.S.M. (2016). Multi-trait QTL analysis for agronomic and quality characters of *Agaricus bisporus* (button mushrooms). AMB Expr..

