# Supplementary material for: Growth-Rate Related Quantitative Trait Locus Analysis of Monokaryotic Isolates of Grifola albicans f. huishuhua (Maitake)
Source: J Fungi (Basel). 2025 Dec 5;11(12):865. doi: 10.3390/jof11120865 (PMC12734058; doi:10.3390/jof11120865)
Supplement: Supplementary file 1 [file jof-11-00865-s001.zip › Supplementary Table.pdf]

Growth-rate related quantitative trait locus analysis of monokaryotic isolates of *Grifola albicans* f. *huishuhua* (Maitake)

Panpan Zhang [1], Junling Wang [2], Guojie Li [1], Shangshang Xiao [1], Lei Sun [1], Xiao Li [1], Jinghua Tian [1], Ming [1], Shoumian Li [1,*]

1. Key Laboratory of Vegetable Germplasm Innovation and Utilization of Hebei, College of Horticulture, Hebei Agricultural University, Baoding 071001, China
2. College of life Sciences, Hebei Agricultural University, Baoding 071001, China;

* Correspondence: yylsm@hebau.edu.cn

Supplementary Table S1 MGRC statistical analysis of monokaryon mycelium on sawdust medium

Strain number	MGRC (mm/d)		Strain number	MGRC (mm/d)	
72	4.953±0.406	a	38	3.762±0.241	cdefghikl
51	4.871±0.590	ab	71	3.762±0.328	cdefghikl
37	4.747±0.438	ab	35	3.745±0.114	cdefghikl
44	4.516±0.233	ab	25	3.725±0.268	cdefghikl
53	4.406±0.332	abc	11	3.719±0.346	defghijklm
60	4.401±0.192	abc	20	3.704±0.281	defghijklm
54	4.332±0.323	abc	33	3.702±0.186	defghijklm
89	4.331±0.472	abc	59	3.701±0.166	defghijklm
50	4.325±0.319	abc	77	3.700±0.127	defghijklm
42	4.278±0.291	abcd	80	3.676±0.504	defghijklm
74	4.277±0.138	abcd	58	3.672±0.192	defghijklm
65	4.267±0.414	abcd	70	3.662±0.423	defghijklm
85	4.247±0.267	abcd	13	3.660±0.398	defghijklm
40	4.230±0.208	abcd	45	3.649±0.142	defghijklm
55	4.206±0.165	abcd	06	3.631±0.298	defghijklm
79	4.184±0.299	abcde	75	3.623±0.363	defghijklm
69	4.175±0.298	abcde	82	3.605±0.502	defghijklm
88	4.166±0.512	abcdef	09	3.586±0.215	efijklmn
90	4.119±0.290	abcdef	61	3.576±0.159	efijklmn
47	4.116±0.202	abcdef	63	3.570±0.598	efijklmn
29	4.112±0.182	abcdef	12	3.561±0.500	efijklmn
15	4.093±0.125	abcdef	83	3.537±0.207	fghijklmn
46	4.090±0.250	bcdef	24	3.535±0.120	fghijklmn
10	4.084±0.099	bcdef	66	3.521±0.121	fghijklmn
48	4.079±0.190	bcdef	03	3.517±0.100	fghijklmn
Y1-18	4.056±0.286	bcdefg	43	3.487±0.230	fghijklmn
23	4.028±0.352	bcdefg	81	3.484±0.298	ghijklmno
91	4.004±0.278	bcdefg	39	3.399±0.287	hijklmno
86	3.995±0.179	bcdefgh	26	3.390±0.206	hijklmno
92	3.985±0.209	bcdefgh	18	3.384±0.285	hijklmno
21	3.978±0.064	bcdefgh	73	3.375±0.420	hijklmno
76	3.965±0.268	bcdefgh	68	3.349±0.173	hijklmno
62	3.954±0.377	bcdefgh	87	3.335±0.237	hijklmno
67	3.928±0.262	cdefgh	27	3.318±0.232	hijklmnop
52	3.921±0.321	cdefgh	07	3.304±0.199	ijklmnop
02	3.909±0.434	cdefgh	30	3.260±0.271	jklmnop
49	3.904±0.137	cdefgh	84	3.233±0.102	jklmnop
64	3.901±0.193	cdefghi	04	3.227±0.399	jklmnop

01	3.867±0.246	cdefghi	22	3.173±0.281	klmnop
56	3.862±0.166	cdefghi	17	3.171±0.087	klmnop
Q3-8	3.859±0.355	cdefghi	31	3.118±0.182	lmnop
57	3.852±0.372	cdefghi	36	3.087±0.169	mnop
14	3.805±0.163	cdefghij	34	3.049±0.169	nop
28	3.799±0.172	cdefghij	08	2.943±0.238	nop
78	3.798±0.177	cdefghij	32	2.903±0.143	op
41	3.785±0.311	cdefghijk	05	2.809±0.414	p
19	3.768±0.185	cdefghikl	16	2.768±0.107	p

Supplementary Table S2 Growing rate statistical analysis of MGRP of monokaryon mycelium on PDA medium

Strain number	MGRP (mm/d)		Strain number	MGRP (mm/d)	
30	3.620±0.301	a	42	2.644±0.192	fghijklmno
03	3.563±0.281	ab	83	2.613±0.056	fghijklmno
08	3.520±0.228	abc	90	2.586±0.137	ghijklmno
19	3.509±0.271	abc	21	2.564±0.064	ghijklmnop
58	3.425±0.233	abcd	88	2.516±0.231	ghijklmnop
44	3.424±0.188	abcd	77	2.486±0.043	ghijklmnop
47	3.413±0.176	abcd	54	2.480±0.241	ghijklmnop
92	3.403±0.175	abcd	67	2.460±0.014	ghijklmnop
15	3.383±0.205	abcd	45	2.440±0.128	hijklmnop
78	3.365±0.264	abcde	56	2.439±0.098	hijklmnopq
02	3.333±0.303	abcde	86	2.421±0.065	hijklmnopq
39	3.318±0.271	abcde	74	2.405±0.113	ijklmnopq
53	3.315±0.125	abcde	46	2.389±0.387	ijklmnopq
64	3.310±0.399	abcde	41	2.386±0.290	ijklmnopq
91	3.264±0.158	abcdef	79	2.383±0.079	ijklmnopq
55	3.234±0.062	abcdef	89	2.349±0.087	jklmnopq
75	3.196±0.106	abcdef	29	2.325±0.383	jklmnopq
37	3.186±0.412	abcdef	Q3-8	2.294±0.382	jklmnopq
57	3.185±0.347	abcdef	84	2.281±0.139	jklmnopq
48	3.182±0.312	abcdef	25	2.268±0.156	klmnopq
13	3.129±0.194	abcdef	27	2.266±0.058	klmnopq
65	3.128±0.393	abcdef	80	2.240±0.213	klmnopq
17	3.092±0.274	abcdefg	23	2.212±0.100	lmnopq
72	3.032±0.246	bcdefg	18	2.187±0.201	lmnopq
26	3.028±0.257	bcdefg	32	2.178±0.096	lmnopq
09	3.019±0.122	cdefg	43	2.174±0.117	lmnopq
20	2.993±0.165	cdefg	60	2.166±0.225	lmnopq
50	2.991±0.278	cdefg	69	2.159±0.206	mnopqr
35	2.985±0.183	cdefgh	10	2.157±0.242	mnopqr

04	2.980±0.128	cdefgh	81	2.153±0.146	mnopqr
Y1-18	2.978±0.265	cdefgh	01	2.137±0.165	nopqr
85	2.946±0.172	cdefgh	51	2.126±0.353	nopqr
33	2.885±0.191	defghi	82	2.100±0.102	opqr
07	2.885±0.216	defghi	14	2.100±0.128	opqr
11	2.824±0.184	efghij	70	2.067±0.361	opqr
36	2.765±0.156	fghijk	22	2.038±0.174	pqr
34	2.756±0.116	fghijkl	16	2.008±0.153	qr
76	2.754±0.183	fghijkl	28	2.005±0.131	qr
38	2.739±0.216	fghijkl	66	1.991±0.182	qr
68	2.721±0.404	fghijkl	71	1.984±0.131	qr
59	2.718±0.400	fghijkl	62	1.977±0.415	qr
24	2.697±0.213	fghijklm	31	1.937±0.127	qr
40	2.688±0.397	fghijklm	87	1.928±0.025	qr
49	2.680±0.255	fghijklm	61	1.914±0.098	qr
06	2.674±0.135	fghijklmn	52	1.788±0.141	qr
05	2.659±0.188	fghijklmn	63	1.727±0.381	qr
73	2.656±0.225	fghijklmn	12	1.649±0.107	r
